# Impact of host genetic polymorphisms on response to inactivated influenza vaccine in children

**DOI:** 10.1038/s41541-023-00621-1

**Published:** 2023-02-20

**Authors:** Tim K. Tsang, Can Wang, Nicole N. Y. Tsang, Vicky J. Fang, Ranawaka A. P. M. Perera, J. S. Malik Peiris, Gabriel M. Leung, Benjamin J. Cowling, Dennis K. M. Ip

**Affiliations:** 1 WHO Collaborating Centre for Infectious Disease Epidemiology and Control, School of Public Health, Li Ka Shing Faculty of Medicine, The University of Hong Kong, Hong Kong Special Administrative Region, China; 2 Laboratory of Data Discovery for Health Limited, Hong Kong Science and Technology Park, Hong Kong Special Administrative Region, China; 3 HKU-Pasteur Research Pole, The University of Hong Kong, Hong Kong Special Administrative Region, China

**Keywords:** Influenza virus, Epidemiology

## Abstract

In randomized controlled trials of influenza vaccination, 550 children received trivalent-inactivated influenza vaccine, permitting us to explore relationship between vaccine response and host single nucleotide polymorphisms (SNPs) in 23 candidate genes with adjustment of multiple testing. For host SNPs in TLR7–1817G/T (rs5741880), genotype GT was associated with lower odds (OR: 0.22, 95% CI: 0.09, 0.53) of have post-vaccination hemagglutination-inhibiting (HAI) titers ≥40, compared with genotype GG and TT combined under the over-dominant model. For host SNPs in TLR8–129G/C (rs3764879), genotype GT was associated with lower odds (OR: 0.47; 95% CI: 0.28, 0.80) of have post vaccination HAI titers ≥40 compared with genotype GG and AA combined under the over-dominant model. Our results could contribute to the development of better vaccines that may offer improved protection to all recipients.

## Main

Influenza vaccination is one of the most effective strategies to control infection and transmission in community. However, there are some vaccinated people, such as elderly, and vary widely among others, with only limited immune responses and hence protection^1–3^. Comparing to other better-known determinants such as the degree of matching and various viral and host factors, host genetic make-up as a potential factor affecting immune responses after vaccination is less explored^4–8^. Currently, the field of vaccinology is still empirical in several aspects, and hence it remains difficult to understand poor vaccine immunogenicity in different pathological and physiological conditions^9^. Similar to other healthcare fields, a personalized approach is proposed to the practice of vaccinology^9–11^. Determining the host factors of immune response induced by influenza vaccine could contribute to development of new personalized vaccines, and new patient-oriented vaccination strategies^6,12^. Enhancing the understanding on genetic determinants of vaccine responses may help to identify individuals with potential of poor vaccine response for guiding more personalized vaccine development and targeted vaccination strategy.

In particular, there is scarcity of data about this topic in the pediatric population^13–15^. It is necessary to direct research toward the production of evidence related to vaccine response in the pediatric age, also in light of the important economic and social burden linked to influenza in this target population^16^.

Host polymorphisms has shown to be associated with immune response after vaccination^17^, vaccine-related adverse events^17^, and disease severity^18^ of various infectious diseases. Major examples included the association of polymorphisms in mannose-binding lectin (MBL)–2 gene encodes a calcium-dependent protein which is important for innate immunity, and associated with increased susceptibility to several infections^19,20^. Several polymorphisms in promoter regions in Interleukin (IL)–10 is associated with the regulation of cellular immune responses^21,22^, Toll-like receptor (TLR) gene with innate immune responses trigging^23,24^ and disease severity^25^. Polymorphism of genes involved in membrane trafficking and antigen processing, and was reported to have significant impact on human response to influenza vaccination^26^.

Here, we analyze the data of immune response and adverse response in two randomized placebo-controlled trials in influenza vaccination in children in Hong Kong^2,3^, to explore the relationship between host single nucleotide polymorphisms (SNPs) and immune responses, measured by post-vaccination hemagglutination-inhibiting (HAI) titers.

In these two trials, there were 550 children were recruited and randomized to receive TIV. After excluding 15–18 children with missing vaccine response with different definitions, and 50–82 children with missing genotype information (depending on the gene), 450–485 children were included in each analysis. Among those vaccinated children, 376/535 (70.3%) of them were classified as responder based on having post-vaccination titer ≥40 for the three vaccine strains, 181/535 (33.8%) of them were classified as responder based on ≥4-fold rise comparing pre- and post-vaccination titer for the three vaccine strains. The average increase of logarithm of GMT for the three vaccine strains after vaccination was 3.44 (95% confidence interval (CI): 3.28, 3.61) log_2_ GMT.

The frequency of genotypes of the 23 host SNPs were summarized (Supplementary Table 1). Five host SNPs were excluded from further analysis since >99% of participants had the same genotype. Also, two TLR8 SNPs (rs3764880 and rs3764879) had almost the same distribution among our participants, therefore we focused on rs3764880 in the analysis.

We estimated the association between host SNPs and vaccine response by logistic regressions (Supplementary Table 2), under dominant model (Supplementary Table 3), recessive model (Supplementary Table 4), over-dominant model (Supplementary Table 5) and multiplicative model (Supplementary Table 6) for the 18 host SNPs. After adjusting for multiple testing, we identified that SNPs in TLR7 (rs5741880) and TLR8 (rs3764880) were associated with vaccine response. For host SNPs in TLR7 (Table 1), genotype GT was associated with lower odds (OR: 0.22, 95% CI: 0.09, 0.53) of have post-vaccination titers ≥40, and 72% (95% CI: 37%, 87%) lower average increase of logarithm of GMT after vaccination for the three vaccine strains, compared with genotype GG and TT combined under the over-dominant model, after adjustment of multiple testing. Consistently, differences in vaccine response among genotype, measured by vaccination titers ≥40, or lower average increase of logarithm of GMT after vaccination for the three vaccine strains, were also detected in dominant model and multiplicative model, although these differences cannot reach statistical significance after multiple testing.

**Table 1:** Relationship between host SNPs in TLR7 (rs5741880) and vaccine response.

Genotype		GG	GT	*TT*	*p* value	Adjusted *p* value
HAI titer ≥1:40 after vaccination for the three vaccine strains (H1N1, H3N2 and B)						
	Proportion ≥1:40	320/463 (69.1%)	8/24 (33.3%)	7/8 (87.5%)		
Dominant model	Odds ratio	1.0	0.39 (0.19,0.81)	0.39 (0.19,0.81)	0.01	0.09
Recessive model	Odds ratio	1.0	1.0	3.39 (0.41,27.82)	0.26	0.69
Over-dominant model	Odds ratio	1.0	0.22 (0.09,0.53)	1.0	0.001	0.02
multiplicative model	Odds ratio	1.0	0.66 (0.39,1.15)	0.44 (0.15,1.32)	0.14	0.40
Average increase of logarithm of geometric mean titer (GMT) after vaccination for the three vaccine strains (H1N1, H3N2 and B)						
	Average increase of logarithm of GMT	3.53 (3.36,3.71)	2.26 (1.58,2.95)	2.71 (1.02,4.39)		
Dominant model	Relative increase	1.0	0.31 (0.16,0.63)	0.31 (0.16,0.63)	0.001	0.02
Recessive model	Relative increase	1.0	1.0	0.47 (0.12,1.82)	0.27	0.84
Over-dominant model	Relative increase	1.0	0.28 (0.13,0.63)	1.0	0.002	0.03
multiplicative model	Relative increase	1.0	0.46 (0.27,0.78)	0.21 (0.08,0.61)	0.004	0.07
≥4-fold rise in antibody after vaccination for the three vaccine strains (H1N1, H3N2 and B)						
	Proportion of 4-fold or greater rise	146/460 (31.7%)	3/24 (12.5%)	3/8 (37.5%)		
Dominant model	Odds ratio	1.0	0.50 (0.20,1.23)	0.50 (0.20,1.23)	0.13	0.58
Recessive model	Odds ratio	1.0	1.0	1.35 (0.32,5.72)	0.69	0.90
Over-dominant model	Odds ratio	1.0	0.31(0.09,1.04)	1.0	0.06	0.31
multiplicative model	Odds ratio	1.0	0.72 (0.37,1.38)	0.52 (0.14,1.91)	0.32	0.83

Adjusted *p* value were obtained from Benjamini-Hochberg Procedure to account for multiple testing.

For host SNPs in TLR8 (Table 2), genotype GA was associated with lower odds (OR: 0.47; 95% CI: 0.28, 0.80) of have post vaccination titers ≥40 compared with genotype GG and AA combined under the over-dominant model, after adjustment of multiple testing. Differences in vaccine response for these two definitions were also detected for dominant model, but could not reach statistical significance after adjusting for multiple testing.

**Table 2:** Relationship between host SNPs in TLR8 (rs3764880) and vaccine response.

Genotype		GG	GA	*AA*	*p* value	Adjusted *p* value
HAI titer ≥1:40 after vaccination for the three vaccine strains (H1N1, H3N2 and B)						
	Proportion ≥1:40	270/383 (70.5%)	34/65 (52.3%)	30/46 (65.2%)		
Dominant model	Odds ratio	1.0	0.57 (0.37,0.88)	0.57 (0.37,0.88)	0.01	0.09
Recessive model	Odds ratio	1.0	1.0	0.89 (0.47,1.68)	0.72	0.87
Over-dominant model	Odds ratio	1.0	0.47 (0.28,0.80)	1.0	0.005	0.03
multiplicative model	Odds ratio	1.0	0.77 (0.58,1.02)	0.59 (0.33,1.04)	0.07	0.34
Average increase of logarithm of geometric mean titer (GMT) after vaccination for the three vaccine strains (H1N1, H3N2 and B)						
	Average increase of logarithm of GMT	3.55 (3.35,3.74)	2.91 (2.44,3.39)	3.44(2.83,4.04)		
Dominant model	Relative increase	1.0	0.66 (0.44,0.99)	0.66 (0.44,0.99)	0.047	0.27
Recessive model	Relative increase	1.0	1.0	0.98 (0.54,1.79)	0.96	0.98
Over-dominant model	Relative increase	1.0	0.54 (0.32,0.89)	1.0	0.02	0.09
multiplicative model	Relative increase	1.0	0.83 (0.63,1.09)	0.69 (0.40,1.19)	0.18	0.55
≥4-fold rise in antibody after vaccination for the three vaccine strains (H1N1, H3N2 and B)						
	Proportion of 4-fold or greater rise	123/381 (32.3%)	12/65 (18.5%)	16/45 (35.6%)		
Dominant model	Odds ratio	1.0	0.72 (0.44,1.16)	0.72 (0.44,1.16)	0.17	0.58
Recessive model	Odds ratio	1.0	1.0	1.27 (0.67,2.42)	0.47	0.88
Over-dominant model	Odds ratio	1.0	0.47 (0.24,0.90)	1.0	0.02	0.20
multiplicative model	Odds ratio	1.0	0.91 (0.67,1.25)	0.84 (0.45,1.55)	0.57	0.83

Adjusted *p* value were obtained from Benjamini-Hochberg Procedure to account for multiple testing.

No serious post-vaccination response including anaphylaxis or shock was reported by any recipient. We found no association between host SNPs and vaccine adverse responses, defined as presence of more than two symptoms, after adjusting for multiple testing (Supplementary Table 7). Before adjusting for multiple testing, we found that host SNPs in IL-6 (rs1818879) was associated with vaccine adverse response under the dominant and over-dominant model. Host SNPs in CCL1 (rs2282691) were associated with vaccine adverse response under the over-dominant model.

Immune response of influenza vaccination is showed to be heterogeneous, despite constant vaccine formulation, possibility due to both host factors like vaccine history^27^, and viral factors like mutation rate of influenza virus^28^. Here, we examined the relationship between variation in the host genetic make-up and the immune response of influenza vaccination, based on a sample of 550 individuals that participated in two large-scale clinical trials of influenza vaccination. We found a significant association between host SNPs in TLR7 and TLR 8 gene on immune response to vaccination.

The TLR family is important for activation of innate immunity and pathogen recognition^29^. Various TLRs exhibit different patterns of expression. Animal studies show that TLR7 is associated with pathology of influenza A virus infection^30^. TLR7 is showed to be a vital component of antiviral immunity^31^ and play an important role of triggering the immune response of COVID-19^32^. TLR8 is showed to madidate reversal of CD4 + regulatory T cell function^33^ and linked with the susceptibility of pulmonary tuberculosis^34^. TLR7–8 recognizes single-stranded RNA virus including influenza^35,36^ and HIV. TLR7–8 agonists can enhance activation of innate immune cells such as CD8 + T cell responses^23,24^, and are suggested to be vaccine adjuvants^37^. Specifically, TLR7 encodes pattern-recognition receptors that regulate immune responses acting as viral RNA sensors, is strongly activated only in symptomatic subjects. This unique transcriptional signature manifests 36 h before peak symptoms and is predictive of disease severity^38^. TLR7 and TLR8 involving in viral sensing play a central role in the vaccine response to trivalent influenza vaccine (TIV) in adults within 24 h after immunization^39^. While TLR polymorphism has been reported to be associated with increase influenza virus A replication, its pathogenicity, and fatality^40^, its association with influenza vaccine response has not been reported in previous studies^17,40^. Our results illustrate the potential role of TLR7–8 gene as key regulators in immunogenicity of seasonal influenza vaccine.

There are limitations in our study. First, the host SNPs but not the entire genome was assessed. Second, the sample size was insufficient to properly account for multiple testing and may only allow detection of large effect associated with host SNPs. Third, further exploration to include other SNPs is needed, such as the SNPs recently reported to affect disease severity (rs12252-C IFITM3)^41^; rs1755609, rs2438409 GLDC)^41,42^ and influenza vaccination (rs12252-C IFITM3; rs743811 HO-1, rs2160567 HO-2; rs10220412 IGHV1–69; rs8099917 IL-28B; rs17793951, rs1175540, rs2972164 PPARG, rs2071045 LEP, rs876537 CRP; HLA gene polymorphism)^7,43–47^. Interplay between different gene polymorphism and humoral response^48^, the immunogenetics of different influenza vaccines^49^, and the induced immune response against evolving influenza virus, and the mechanism of influenza-host genetic interactions may be explored in future studies. Fourth, our sample size may be underpowered in some genetic models (Supplementary Table 8), and hence there could be some SNPs that could have effect on influenza vaccine response, but unidentified. Finally, there could be difference in vaccine response among strains (Supplementary Table 9), and such heterogeneity may reduce the power to detect association.

In conclusion, the result from this study has demonstrated the importance of host genetic variation in affecting the response to influenza vaccination. Our findings may help to explain the great variability in the protection achieved by influenza vaccination in different individuals. The identification of genetic variations associated with poor response and adverse effect on receiving influenza vaccination also enhanced our understanding in the area and could contribute to the development of better vaccines that may offer improved protection to all recipients. Our study could help to overcome barriers in the field of vaccinology and the response of vaccines, particularly for pediatric population. Our study also provides a framework how influenza vaccines can be optimized by considering immunogenetics in its design, including the exploration on adjuvants that target the proteins encoded by these TLR genes to circumvent immunogenetic restrictions.

## Methods

### Study design

Data were collected in two community-based randomized controlled trials of influenza vaccination conducted in 2008–2009 (pilot study) and 2009–2010 (main study) in Hong Kong^2,3^. In these trials, children (6–15 y in pilot study and 6–17 y in main study) were randomly allocated to receive either a single dose of trivalent-inactivated influenza vaccine (TIV, Sanofi Pasteur) or saline placebo. Serum specimens were collected at the enrollment to the study and 1 month after vaccination.

### Ethics

Proxy written consent from parents or legal guardians was obtained for participants who were <18 years old, with additional written assent from those ages 8–17 years. The study protocol was approved by the Institutional Review Board of the University of Hong Kong.

### Laboratory methods

Serum specimens were tested against the vaccine strains A/Brisbane/59/2007(H1N1) and B/Brisbane/60/2008-like (Victoria lineage), the prevalent seasonal strain A/Perth/16/2009-like(H3N2), in parallel by hemagglutination inhibition assays in serial doubling dilutions from an initial dilution of 1:10 using standard methods^50^.

Whole blood samples were collected for genetic analysis in this study. DNA was extracted and genotyped for SNPs for IL-1B -511G > A (rs16944), IL-6–5843A/G (rs1818879), IL-8–251T/A (rs4073), IL-10–1082T/C (rs1800896), -819A/G (rs1800871), -592T/G (rs1800872), MBL-2–5232C > T, (rs1800451). -221C/G (rs7096206), -34G > A (rs5030737). -550G > C (rs11003125), MxA-88G/T (rs2071430), OAS1–347A/G (rs2660), RIG1 G/C (rs9695310), TLR3–1377T/G (rs3775290), -7C/A (rs3775296), TLR4 G/A (rs5030718), Asp299Gly (rs4986790), TLR7 Gln11Leu (rs179008), 1817G/T (rs5741880), TLR8–129G/C (rs3764879), Met1Val (rs3764880), and (rs11003131)G/T. These SNPs were selected based on a candidate gene approach, selected based on previous literatures^17–25^.

### Statistical analysis

We measured the response to influenza vaccine by (1) post-vaccination titers ≥1:40 for the three vaccine strains, (2) ≥4-fold rise after vaccination for the three vaccine strains and (3) the average increase of logarithm of geometric mean titers (GMT) for the three vaccine strains. To evaluate the relationship between host SNPs with vaccine response, we tested four genetic models, including dominant model, recessive model, over-dominant model and multiplicative models^51^. Therefore, for each host SNPs, genotypes were combined under different genetic models in the statistical analysis. In each genetic model, we used logistic regression to estimate the odds ratio of antibody response to vaccination among different genotype. We used the Benjamini-Hochberg Procedure to adjust the *p* value for multiple testing^52^. The same procedure was repeated to explore the relationship between adverse vaccine response, defined as presence of two or more symptoms out of ten following symptoms within 4 days after vaccination: fever, chills, fatigue, headache, cough, muscle pain, swell, redness, bruising and injection pain. All statistical analyses were conducted using R version 3.6.1 (R Foundation for Statistical Computing, Vienna, Austria).

### Reporting summary

Further information on research design is available in the Nature Portfolio Reporting Summary linked to this article.

## Supplementary information

Supplemental material

Reporting summary

## Data Availability

The data that support the findings of this study are available on request from the corresponding author T.K.T. The data are not publicly available due to containing information that could compromise research participant privacy.
