# Supplementary material for: Impact of host genetic polymorphisms on response to inactivated influenza vaccine in children
Source: NPJ Vaccines. 2023 Feb 20;8:21. doi: 10.1038/s41541-023-00621-1 (PMC9940051; doi:10.1038/s41541-023-00621-1)
Supplement: Supplementary file 1 — Supplemental material [file 41541_2023_621_MOESM1_ESM.docx]

**Supplementary Material**

**for ‘Impact of host genetic polymorphisms on response to inactivated influenza vaccine in children”**

Tim K. Tsang^1,2^, Can Wang^1^, Nicole N.Y. Tsang^1^, Vicky J. Fang^1^, Ranawaka A. P. M. Perera^1^, J. S. Malik Peiris^1,3^, Gabriel M. Leung^1,2^, Benjamin J. Cowling^1,2^, Dennis K. M. Ip^1^

**Affiliations:**

1. WHO Collaborating Centre for Infectious Disease Epidemiology and Control, School of Public Health, Li Ka Shing Faculty of Medicine, The University of Hong Kong, Hong Kong Special Administrative Region, China.

2. Laboratory of Data Discovery for Health Limited, Hong Kong Science and Technology Park, New Territories, Hong Kong.

3. HKU-Pasteur Research Pole, The University of Hong Kong, Hong Kong Special Administrative Region, China.

**Supplementary Table 1**. Genotype frequency of host SNPs

| Rs number | Gene | Genotype 1 | Frequency | Genotype 2 | Frequency | Genotype 3 | Frequency | Missing values |
| --- | --- | --- | --- | --- | --- | --- | --- | --- |
| rs1818879 | IL-6 | AA | 280 (59.6%) | AG | 187 (39.8%) | GG | 3 (0.6%) | 80 |
| rs1800451 | MBL-2 | CC | 499 (100%) | TC | 0 (0%) | TT | 0 (0%) | 51 |
| rs2660 | OAS1 | AA | 297 (59.6%) | GA | 174 (34.9%) | GG | 27 (5.4%) | 52 |
| rs2282691 | CCL1 | TT | 217 (43.5%) | AT | 210 (42.1%) | AA | 72 (14.4%) | 51 |
| rs1800896 | IL-10 | TT | 453 (90.8%) | CT | 46 (9.2%) | CC | 0 (0%) | 51 |
| rs3764879 | TLR8 | GG | 387 (77.6%) | CG | 67 (13.4%) | CC | 45 (9%) | 51 |
| rs3775290 | TLR3 | TT | 188 (37.7%) | GT | 233 (46.7%) | TT | 78 (15.6%) | 51 |
| rs1800871 | IL-10 | AA | 239 (51.1%) | GA | 179 (38.2%) | GG | 50 (10.7%) | 82 |
| rs3764880 | TLR8 | GG | 386 (77.4%) | GA | 67 (13.4%) | AA | 46 (9.2%) | 51 |
| rs2071430 | MxA | GG | 261 (52.4%) | GT | 196 (39.4%) | TT | 41 (8.2%) | 52 |
| rs5030718 | TLR4 | GG | 494 (99%) | GA | 5 (1%) | AA | 0 (0%) | 51 |
| rs7096206 | MBL-2 | CC | 334 (67.1%) | CG | 142 (28.5%) | GG | 22 (4.4%) | 52 |
| rs1800872 | IL-10 | TT | 239 (47.9%) | GT | 210 (42.1%) | GG | 50 (10%) | 51 |
| rs179008 | TLR7 | AA | 499 (100%) | AT | 0 (0%) | TT | 0 (0%) | 51 |
| rs4986790 | TLR4 | AA | 499 (100%) | AG | 0 (0%) | GG | 0 (0%) | 51 |
| rs4073 | IL8 | TT | 180 (36.1%) | TA | 225 (45.1%) | AA | 94 (18.8%) | 51 |
| rs9695310 | RIG-I | GG | 205 (43.1%) | GC | 223 (46.8%) | CC | 48 (10.1%) | 74 |
| rs3775296 | TLR3 | CC | 302 (60.4%) | CA | 160 (32%) | AA | 38 (7.6%) | 50 |
| rs16944 | IL-1B | GG | 133 (26.6%) | AG | 257 (51.4%) | AA | 110 (22%) | 50 |
| rs5030737 | MBL-2 | GG | 500 (100%) | GA | 0 (0%) | AA | 0 (0%) | 50 |
| rs11003125 | MBL-2 | GG | 153 (30.6%) | CG | 255 (51%) | CC | 92 (18.4%) | 50 |
| rs11003131 | TLR8 | GG | 352 (70.4%) | GT | 137 (27.4%) | TT | 11 (2.2%) | 50 |
| rs5741880 | TLR7 | GG | 468 (93.6%) | GT | 24 (4.8%) | TT | 8 (1.6%) | 50 |

**Supplementary Table 2**. Genetic models used in the analysis (0 indicates reference group)

| Genetic model | Value in the model | | | Statistical analysis |
| --- | --- | --- | --- | --- |
| Genotype | AA | Aa | aa |  |
| Dominant | 0 | 1 | 1 | Logistic regression |
| Recessive | 0 | 0 | 1 | Logistic regression |
| Over-dominant | 0 | 1 | 0 | Logistic regression |
| Multiplicative | 0 | 1 | 2 | Linear regression |

**Supplementary Table 3.** Relationship between host SNPs and vaccine response under dominant model. Adjusted p-value were obtained from Benjamini-Hochberg Procedure to account for multiple testing.

| Definition of vaccine response | | HAI titer ≥1:40 after vaccination for the three vaccine strains (H1N1, H3N2 and B) | | | Average increase of logarithm of GMT after vaccination for the three vaccine strains (H1N1, H3N2 and B) | | | 4-fold or greater rise in antibody after vaccination for the three vaccine strains (H1N1, H3N2 and B) | | |
| --- | --- | --- | --- | --- | --- | --- | --- | --- | --- | --- |
| Rs number | Gene | Odds ratio | p-value | Adjusted p-value | Relative increase | p-value | Adjusted p-value | Odds ratio | p-value | Adjusted p-value |
| rs1818879 | IL-6 | 1.13 (0.76,1.68) | 0.55 | 0.85 | 0.84  (0.58,1.21) | 0.34 | 0.68 | 0.87 (0.58,1.30) | 0.49 | 0.77 |
| rs1800451 | MBL-2 |  |  |  |  |  |  |  |  |  |
| rs2660 | OAS1 | 1.16 (0.79,1.71) | 0.44 | 0.76 | 1.16  (0.81,1.64) | 0.42 | 0.68 | 1.19 (0.81,1.76) | 0.38 | 0.77 |
| rs2282691 | CCL1 | 1.32 (0.90,1.92) | 0.15 | 0.37 | 1.11  (0.78,1.57) | 0.56 | 0.68 | 1.07 (0.73,1.58) | 0.72 | 0.82 |
| rs1800896 | IL-10 |  |  |  |  |  |  |  |  |  |
| rs3764879 | TLR8 | 0.59 (0.38,0.91) | 0.02* | 0.096 | 0.66  (0.43,0.99) | 0.047* | 0.27 | 0.73 (0.45,1.18) | 0.20 | 0.58 |
| rs3775290 | TLR3 | 1.04 (0.71,1.54) | 0.83 | 0.996 | 1.29  (0.90,1.83) | 0.17 | 0.47 | 1.09 (0.74,1.63) | 0.66 | 0.81 |
| rs1800871 | IL-10 | 1.01 (0.68,1.49) | 0.97 | 0.996 | 0.98  (0.69,1.40) | 0.92 | 0.96 | 1.15 (0.77,1.70) | 0.50 | 0.77 |
| rs3764880 | TLR8 | 0.57 (0.37,0.88) | 0.01* | 0.09 | 0.66  (0.44,0.99) | 0.047* | 0.27 | 0.72 (0.44,1.16) | 0.17 | 0.58 |
| rs2071430 | MxA | 1.17 (0.80,1.71) | 0.41 | 0.76 | 0.90  (0.64,1.27) | 0.55 | 0.68 | 1.05 (0.72,1.55) | 0.79 | 0.84 |
| rs5030718 | TLR4 |  |  |  |  |  |  |  |  |  |
| rs7096206 | MBL-2 | 1.38 (0.92,2.09) | 0.12 | 0.35 | 1.14  (0.79,1.64) | 0.49 | 0.68 | 1.09 (0.73,1.64) | 0.67 | 0.81 |
| rs1800872 | IL-10 | 1.01 (0.69,1.47) | 0.96 | 0.996 | 1.01  (0.71,1.42) | 0.96 | 0.96 | 1.15 (0.78,1.68) | 0.48 | 0.77 |
| rs179008 | TLR7 |  |  |  |  |  |  |  |  |  |
| rs4986790 | TLR4 |  |  |  |  |  |  |  |  |  |
| rs4073 | IL8 | 1.00 (0.68,1.48) | 0.996 | 0.996 | 0.77  (0.54,1.11) | 0.16 | 0.47 | 0.81 (0.54,1.20) | 0.29 | 0.69 |
| rs9695310 | RIG-I | 1.39 (0.94,2.04) | 0.10 | 0.33 | 1.31  (0.92,1.88) | 0.14 | 0.47 | 1.30 (0.87,1.93) | 0.20 | 0.58 |
| rs3775296 | TLR3 | 1.20 (0.81,1.77) | 0.36 | 0.76 | 0.88  (0.62,1.25) | 0.47 | 0.68 | 1.02 (0.69,1.50) | 0.93 | 0.93 |
| rs16944 | IL-1B | 1.61 (1.06,2.43) | 0.03* | 0.11 | 1.08  (0.73,1.59) | 0.70 | 0.79 | 0.90 (0.59,1.38) | 0.63 | 0.81 |
| rs5030737 | MBL-2 |  |  |  |  |  |  |  |  |  |
| rs11003125 | MBL-2 | 0.97 (0.64,1.45) | 0.87 | 0.996 | 1.15  (0.79,1.67) | 0.46 | 0.68 | 1.55 (1.00,2.39) | 0.049* | 0.58 |
| rs11003131 | TLR8 | 0.90 (0.60,1.35) | 0.60 | 0.85 | 0.83  (0.57,1.21) | 0.34 | 0.68 | 0.67 (0.43,1.03) | 0.07 | 0.58 |
| rs5741880 | TLR7 | 0.39 (0.19,0.81) | 0.01* | 0.09 | 0.31  (0.16,0.63) | 0.001* | 0.02* | 0.50 (0.20,1.23) | 0.13 | 0.58 |

**Supplementary Table 4.** Relationship between host SNPs and vaccine response under recessive model. Adjusted p-value were obtained from Benjamini-Hochberg Procedure to account for multiple testing.

| Definition of vaccine response | | HAI titer ≥1:40 after vaccination for the three vaccine strains (H1N1, H3N2 and B) | | | Average increase of logarithm of GMT after vaccination for the three vaccine strains (H1N1, H3N2 and B) | | | 4-fold or greater rise in antibody after vaccination for the three vaccine strains (H1N1, H3N2 and B) | | |
| --- | --- | --- | --- | --- | --- | --- | --- | --- | --- | --- |
| Rs number | Gene | Odds ratio | p-value | Adjusted p-value | Relative increase | p-value | Adjusted p-value | Odds ratio | p-value | Adjusted p-value |
| rs1818879 | IL-6 | 0.23 (0.02,2.59) | 0.24 | 0.69 | 1.74 (0.19,16.02) | 0.63 | 0.85 | 0  0 (0,Inf) | 0.98 | 0.98 |
| rs1800451 | MBL-2 |  |  |  |  |  |  |  |  |  |
| rs2660 | OAS1 | 1.40 (0.58,3.37) | 0.46 | 0.78 | 1.51  (0.71,3.22) | 0.29 | 0.84 | 1.60 (0.73,3.54) | 0.24 | 0.85 |
| rs2282691 | CCL1 | 0.93 (0.54,1.58) | 0.78 | 0.89 | 0.67  (0.41,1.09) | 0.11 | 0.84 | 0.69 (0.38,1.23) | 0.21 | 0.85 |
| rs1800896 | IL-10 |  |  |  |  |  |  |  |  |  |
| rs3764879 | TLR8 | 0.95 (0.50,1.83) | 0.7 | 0.94 | 0.99  (0.54,1.80) | 0.96 | 0.98 | 1.32 (0.69,2.52) | 0.40 | 0.85 |
| rs3775290 | TLR3 | 0.79 (0.48,1.32) | 0.37 | 0.70 | 1.25  (0.78,2.01) | 0.36 | 0.84 | 0.60 (0.34,1.08) | 0.09 | 0.74 |
| rs1800871 | IL-10 | 0.89 (0.48,1.66) | 0.72 | 0.87 | 0.80  (0.45,1.42) | 0.45 | 0.84 | 0.89 (0.47,1.72) | 0.74 | 0.90 |
| rs3764880 | TLR8 | 0.89 (0.47,1.68) | 0.72 | 0.87 | 0.98  (0.54,1.79) | 0.96 | 0.98 | 1.27 (0.67,2.42) | 0.47 | 0.88 |
| rs2071430 | MxA | 0.88 (0.45,1.74) | 0.72 | 0.87 | 1.01  (0.54,1.90) | 0.98 | 0.98 | 0.97 (0.48,1.96) | 0.93 | 0.98 |
| rs5030718 | TLR4 |  |  |  |  |  |  |  |  |  |
| rs7096206 | MBL-2 | 1.67 (0.60,4.60) | 0.32 | 0.69 | 1.23  (0.53,2.83) | 0.63 | 0.85 | 1.30 (0.53,3.16) | 0.57 | 0.90 |
| rs1800872 | IL-10 | 0.89 (0.48,1.66) | 0.72 | 0.87 | 0.79  (0.44,1.40) | 0.42 | 0.84 | 0.89 (0.46,1.71) | 0.73 | 0.90 |
| rs179008 | TLR7 |  |  |  |  |  |  |  |  |  |
| rs4986790 | TLR4 |  |  |  |  |  |  |  |  |  |
| rs4073 | IL8 | 0.78 (0.49,1.25) | 0.30 | 0.69 | 0.89  (0.57,1.39) | 0.61 | 0.85 | 0.98 (0.60,1.61) | 0.94 | 0.98 |
| rs9695310 | RIG-I | 2.00 (0.97,4.13) | 0.06 | 0.51 | 0.87  (0.48,1.58) | 0.65 | 0.85 | 0.73 (0.37,1.46) | 0.38 | 0.85 |
| rs3775296 | TLR3 | 1.59 (0.73,3.44) | 0.24 | 0.69 | 0.72  (0.38,1.36) | 0.31 | 0.84 | 1.18 (0.59,2.37) | 0.65 | 0.90 |
| rs16944 | IL-1B | 1.36 (0.85,2.17) | 0.20 | 0.69 | 1.02  (0.68,1.55) | 0.91 | 0.98 | 0.80 (0.50,1.28) | 0.35 | 0.85 |
| rs5030737 | MBL-2 |  |  |  |  |  |  |  |  |  |
| rs11003125 | MBL-2 | 1.01 (0.62,1.64) | 0.98 | 0.98 | 1.58  (1.01,2.46) | 0.045* | 0.77 | 1.55 (0.96,2.49) | 0.07 | 0.74 |
| rs11003131 | TLR8 | 0.26 (0.08,0.92) | 0.04* | 0.51 | 0.45  (0.14,1.43) | 0.17 | 0.84 | 0.49 (0.10,2.30) | 0.37 | 0.85 |
| rs5741880 | TLR7 | 3.39 (0.41,27.82) | 0.26 | 0.69 | 0.47  (0.12,1.82) | 0.27 | 0.84 | 1.35 (0.32,5.72) | 0.69 | 0.90 |

**Supplementary Table 5.** Relationship between host SNPs and vaccine response under over-dominant model. Adjusted p-value were obtained from Benjamini-Hochberg Procedure to account for multiple testing.

| Definition of vaccine response | | HAI titer ≥1:40 after vaccination for the three vaccine strains (H1N1, H3N2 and B) | | | Average increase of logarithm of GMT after vaccination for the three vaccine strains (H1N1, H3N2 and B) | | | 4-fold or greater rise in antibody after vaccination for the three vaccine strains (H1N1, H3N2 and B) | | |
| --- | --- | --- | --- | --- | --- | --- | --- | --- | --- | --- |
| Rs number | Gene | Odds ratio | p-value | Adjusted p-value | Relative increase | p-value | Adjusted p-value | Odds ratio | p-value | Adjusted p-value |
| rs1818879 | IL-6 | 1.18 (0.79,1.76) | 0.42 | 0.72 | 0.83 (0.57,1.19) | 0.30 | 0.80 | 0.90 (0.60,1.35) | 0.62 | 0.85 |
| rs1800451 | MBL-2 |  |  |  |  |  |  |  |  |  |
| rs2660 | OAS1 | 1.09 (0.73,1.62) | 0.67 | 0.86 | 1.06 (0.74,1.53) | 0.75 | 0.85 | 1.07 (0.72,1.60) | 0.73 | 0.85 |
| rs2282691 | CCL1 | 1.38 (0.94,2.03) | 0.10 | 0.44 | 1.36 (0.96,1.93) | 0.09 | 0.29 | 1.28 (0.87,1.88) | 0.21 | 0.51 |
| rs1800896 | IL-10 |  |  |  |  |  |  |  |  |  |
| rs3764879 | TLR8 | 0.47 (0.28,0.80) | 0.005* | 0.03* | 0.54 (0.32,0.89) | 0.02* | 0.09 | 0.47 (0.24,0.90) | 0.02* | 0.20 |
| rs3775290 | TLR3 | 1.18 (0.81,1.73) | 0.39 | 0.72 | 1.13 (0.80,1.59) | 0.49 | 0.80 | 1.39 (0.95,2.04) | 0.09 | 0.32 |
| rs1800871 | IL-10 | 1.06 (0.71,1.58) | 0.78 | 0.86 | 1.07 (0.74,1.55) | 0.71 | 0.85 | 1.21 (0.80,1.81) | 0.36 | 0.62 |
| rs3764880 | TLR8 | 0.47 (0.28,0.80) | 0.005* | 0.03* | 0.54 (0.32,0.89) | 0.02* | 0.09 | 0.47 (0.24,0.90) | 0.02* | 0.20 |
| rs2071430 | MxA | 1.23 (0.83,1.81) | 0.30 | 0.72 | 0.89 (0.63,1.27) | 0.53 | 0.80 | 1.07 (0.72,1.58) | 0.75 | 0.85 |
| rs5030718 | TLR4 |  |  |  |  |  |  |  |  |  |
| rs7096206 | MBL-2 | 1.29 (0.84,1.97) | 0.25 | 0.72 | 1.10 (0.75,1.62) | 0.62 | 0.80 | 1.04 (0.68,1.59) | 0.85 | 0.87 |
| rs1800872 | IL-10 | 1.05 (0.72,1.54) | 0.79 | 0.86 | 1.10 (0.78,1.56) | 0.59 | 0.80 | 1.20 (0.81,1.77) | 0.36 | 0.62 |
| rs179008 | TLR7 |  |  |  |  |  |  |  |  |  |
| rs4986790 | TLR4 |  |  |  |  |  |  |  |  |  |
| rs4073 | IL8 | 1.17 (0.80,1.71) | 0.41 | 0.72 | 0.84 (0.60,1.19) | 0.34 | 0.80 | 0.82 (0.56,1.21) | 0.33 | 0.62 |
| rs9695310 | RIG-I | 1.10 (0.75,1.62) | 0.62 | 0.86 | 1.37 (0.96,1.96) | 0.08 | 0.29 | 1.43 (0.97,2.12) | 0.07 | 0.31 |
| rs3775296 | TLR3 | 1.06 (0.71,1.59) | 0.77 | 0.86 | 0.97 (0.67,1.4) | 0.86 | 0.86 | 0.97 (0.64,1.46) | 0.87 | 0.87 |
| rs16944 | IL-1B | 1.19 (0.82,1.74) | 0.36 | 0.72 | 1.05 (0.74,1.48) | 0.80 | 0.85 | 1.07 (0.73,1.57) | 0.73 | 0.85 |
| rs5030737 | MBL-2 |  |  |  |  |  |  |  |  |  |
| rs11003125 | MBL-2 | 0.97 (0.66,1.41) | 0.86 | 0.86 | 0.86 (0.61,1.21) | 0.39 | 0.80 | 1.09 (0.74,1.59) | 0.68 | 0.85 |
| rs11003131 | TLR8 | 1.05 (0.68,1.60) | 0.84 | 0.86 | 0.90 (0.61,1.33) | 0.59 | 0.80 | 0.70 (0.45,1.10) | 0.12 | 0.34 |
| rs5741880 | TLR7 | 0.22 (0.09,0.53) | 0.001* | 0.02* | 0.28 (0.13,0.63) | 0.002* | 0.03* | 0.31 (0.09,1.04) | 0.06 | 0.31 |

**Supplementary Table 6.** Relationship between host SNPs and vaccine response under multiplicative model. Adjusted p-value were obtained from Benjamini-Hochberg Procedure to account for multiple testing.

| Definition of vaccine response | | HAI titer ≥1:40 after vaccination for the three vaccine strains (H1N1, H3N2 and B) | | | Average increase of logarithm of GMT after vaccination for the three vaccine strains (H1N1, H3N2 and B) | | | 4-fold or greater rise in antibody after vaccination for the three vaccine strains (H1N1, H3N2 and B) | | |
| --- | --- | --- | --- | --- | --- | --- | --- | --- | --- | --- |
| Rs number | Gene | Odds ratio | p-value | Adjusted p-value | Relative increase | p-value | Adjusted p-value | Odds ratio | p-value | Adjusted p-value |
| rs1818879 | IL-6 | 1.08 (0.73,1.59) | 0.711 | 0.91 | 0.86  (0.60,1.22) | 0.40 | 0.62 | 0.84 (0.57,1.25) | 0.39 | 0.83 |
| rs1800451 | MBL-2 |  |  |  |  |  |  |  |  |  |
| rs2660 | OAS1 | 1.16 (0.84,1.60) | 0.36 | 0.65 | 1.17  (0.88,1.56) | 0.29 | 0.61 | 1.21 (0.88,1.65) | 0.24 | 0.83 |
| rs2282691 | CCL1 | 1.13 (0.86,1.48) | 0.39 | 0.65 | 0.95  (0.74,1.22) | 0.70 | 0.76 | 0.95 (0.72,1.25) | 0.71 | 0.83 |
| rs1800896 | IL-10 |  |  |  |  |  |  |  |  |  |
| rs3764879 | TLR8 | 0.79 (0.59,1.05) | 0.10 | 0.34 | 0.83  (0.63,1.09) | 0.18 | 0.55 | 0.93 (0.68,1.27) | 0.63 | 0.83 |
| rs3775290 | TLR3 | 0.96 (0.73,1.26) | 0.75 | 0.91 | 1.20  (0.94,1.54) | 0.15 | 0.55 | 0.92 (0.70,1.21) | 0.56 | 0.83 |
| rs1800871 | IL-10 | 0.98 (0.73,1.31) | 0.89 | 0.92 | 0.94  (0.72,1.23) | 0.67 | 0.76 | 1.05 (0.79,1.41) | 0.73 | 0.83 |
| rs3764880 | TLR8 | 0.77 (0.58,1.02) | 0.07 | 0.34 | 0.83  (0.63,1.09) | 0.18 | 0.55 | 0.91 (0.67,1.25) | 0.57 | 0.83 |
| rs2071430 | MxA | 1.08 (0.80,1.45) | 0.63 | 0.89 | 0.94  (0.72,1.23) | 0.65 | 0.76 | 1.03 (0.76,1.39) | 0.86 | 0.86 |
| rs5030718 | TLR4 |  |  |  |  |  |  |  |  |  |
| rs7096206 | MBL-2 | 1.34 (0.94,1.89) | 0.10 | 0.34 | 1.12  (0.83,1.52) | 0.45 | 0.64 | 1.10 (0.79,1.54) | 0.57 | 0.83 |
| rs1800872 | IL-10 | 0.98 (0.74,1.31) | 0.90 | 0.92 | 0.96  (0.74,1.24) | 0.74 | 0.76 | 1.06 (0.79,1.41) | 0.71 | 0.83 |
| rs179008 | TLR7 |  |  |  |  |  |  |  |  |  |
| rs4986790 | TLR4 |  |  |  |  |  |  |  |  |  |
| rs4073 | IL8 | 0.93 (0.71,1.21) | 0.58 | 0.89 | 0.86  (0.68,1.09) | 0.23 | 0.55 | 0.90 (0.69,1.18) | 0.45 | 0.83 |
| rs9695310 | RIG-I | 1.39 (1.03,1.89) | 0.03* | 0.27 | 1.14  (0.86,1.49) | 0.36 | 0.61 | 1.09 (0.81,1.47) | 0.57 | 0.83 |
| rs3775296 | TLR3 | 1.21 (0.89,1.63) | 0.23 | 0.56 | 0.87  (0.67,1.14) | 0.32 | 0.61 | 1.04 (0.77,1.40) | 0.79 | 0.84 |
| rs16944 | IL-1B | 1.36 (1.03,1.78) | 0.03* | 0.27 | 1.04  (0.81,1.33) | 0.76 | 0.76 | 0.89 (0.67,1.17) | 0.39 | 0.83 |
| rs5030737 | MBL-2 |  |  |  |  |  |  |  |  |  |
| rs11003125 | MBL-2 | 0.99 (0.75,1.30) | 0.92 | 0.92 | 1.23  (0.96,1.58) | 0.11 | 0.55 | 1.40 (1.06,1.85) | 0.02* | 0.34 |
| rs11003131 | TLR8 | 0.81 (0.57,1.17) | 0.27 | 0.57 | 0.81  (0.58,1.13) | 0.21 | 0.55 | 0.68 (0.46,1.02) | 0.06 | 0.51 |
| rs5741880 | TLR7 | 0.66 (0.39,1.15) | 0.14 | 0.40 | 0.46  (0.27,0.78) | 0.004* | 0.07 | 0.72 (0.37,1.38) | 0.32 | 0.83 |

**Supplementary Table 7.** Association between host SNPs and adverse response of vaccination, defined as two or above out of the following symptoms (Fever, chills, fatigue, headache, cough, muscle pain, swell, redness, bruising and injection pain). Adjusted p-value were obtained from Benjamini-Hochberg Procedure to account for multiple testing. NC indicates not calculated due to insufficient sample size.

| Genetic model | | Dominant model | | | Recessive model | | | Over-dominant model | | | Multiplicative model | | |
| --- | --- | --- | --- | --- | --- | --- | --- | --- | --- | --- | --- | --- | --- |
| Rs number | Gene | Odds ratio | p-value | Adjusted p-value | Odds ratio | p-value | Adjusted p-value | Odds ratio | p-value | Adjusted p-value | Odds ratio | p-value | Adjusted p-value |
| rs1818879 | IL-6 | 1.64 (1.05,2.55) | 0.03* | 0.48 | 0  NC | NC | NC | 1.71 (1.10,2.65) | 0.02* | 0.31 | 1.53 (1.00,2.34) | 0.05 | 0.48 |
| rs1800451 | MBL-2 |  |  |  |  |  |  |  |  |  |  |  |  |
| rs2660 | OAS1 | 0.95 (0.62,1.47) | 0.83 | 0.95 | 1.09 (0.45,2.66) | 0.85 | 0.91 | 0.93 (0.60,1.46) | 0.76 | 0.86 | 0.98 (0.69,1.40) | 0.92 | 0.92 |
| rs2282691 | CCL1 | 0.76 (0.5,1.17) | 0.21 | 0.51 | 1.36 (0.75,2.44) | 0.31 | 0.58 | 0.64 (0.41,1.00) | 0.049* | 0.41 | 0.94 (0.69,1.28) | 0.70 | 0.86 |
| rs1800896 | IL-10 |  |  |  |  |  |  |  |  |  |  |  |  |
| rs3764879 | TLR8 | 0.71 (0.41,1.21) | 0.21 | 0.51 | 0.60 (0.26,1.39) | 0.23 | 0.53 | 0.84 (0.44,1.58) | 0.58 | 0.76 | 0.77 (0.53,1.11) | 0.17 | 0.58 |
| rs3775290 | TLR3 | 0.97 (0.63,1.50) | 0.90 | 0.95 | 1.30 (0.74,2.31) | 0.36 | 0.62 | 0.84 (0.55,1.29) | 0.44 | 0.76 | 1.06 (0.78,1.44) | 0.71 | 0.86 |
| rs1800871 | IL-10 | 0.91 (0.59,1.42) | 0.69 | 0.95 | 0.52 (0.23,1.20) | 0.13 | 0.50 | 1.14 (0.73,1.79) | 0.56 | 0.76 | 0.84 (0.60,1.18) | 0.32 | 0.69 |
| rs3764880 | TLR8 | 0.70 (0.41,1.19) | 0.19 | 0.51 | 0.58 (0.25,1.35) | 0.20 | 0.53 | 0.84 (0.44,1.58) | 0.58 | 0.76 | 0.76 (0.53,1.10) | 0.15 | 0.58 |
| rs2071430 | MxA | 1.10 (0.72,1.69) | 0.66 | 0.95 | 1.20 (0.56,2.59) | 0.64 | 0.77 | 1.04 (0.67,1.61) | 0.85 | 0.87 | 1.10 (0.79,1.53) | 0.58 | 0.86 |
| rs5030718 | TLR4 |  |  |  |  |  |  |  |  |  |  |  |  |
| rs7096206 | MBL-2 | 0.73 (0.46,1.17) | 0.19 | 0.51 | 1.10 (0.42,2.89) | 0.84 | 0.91 | 0.69 (0.42,1.14) | 0.15 | 0.62 | 0.82 (0.56,1.21) | 0.32 | 0.69 |
| rs1800872 | IL-10 | 0.96 (0.63,1.47) | 0.84 | 0.95 | 0.51 (0.22,1.18) | 0.12 | 0.50 | 1.18 (0.77,1.81) | 0.44 | 0.76 | 0.86 (0.62,1.20) | 0.38 | 0.72 |
| rs179008 | TLR7 |  |  |  |  |  |  |  |  |  |  |  |  |
| rs4986790 | TLR4 |  |  |  |  |  |  |  |  |  |  |  |  |
| rs4073 | IL8 | 0.87 (0.56,1.36) | 0.55 | 0.94 | 1.51 (0.90,2.54) | 0.12 | 0.50 | 0.67 (0.44,1.04) | 0.07 | 0.41 | 1.07 (0.80,1.44) | 0.65 | 0.86 |
| rs9695310 | RIG-I | 1.03 (0.66,1.59) | 0.90 | 0.95 | 1.47 (0.76,2.84) | 0.25 | 0.53 | 0.88 (0.57,1.36) | 0.56 | 0.76 | 1.11 (0.80,1.54) | 0.52 | 0.86 |
| rs3775296 | TLR3 | 1.42 (0.92,2.18) | 0.11 | 0.51 | 1.81 (0.88,3.73) | 0.11 | 0.50 | 1.18 (0.76,1.85) | 0.46 | 0.76 | 1.37 (0.99,1.89) | 0.06 | 0.48 |
| rs16944 | IL-1B | 0.70 (0.44,1.12) | 0.13 | 0.51 | 0.87 (0.51,1.46) | 0.59 | 0.77 | 0.83 (0.54,1.26) | 0.38 | 0.76 | 0.82 (0.60,1.11) | 0.20 | 0.58 |
| rs5030737 | MBL-2 |  |  |  |  |  |  |  |  |  |  |  |  |
| rs11003125 | MBL-2 | 1.22 (0.76,1.96) | 0.41 | 0.87 | 1.47 (0.87,2.45) | 0.15 | 0.50 | 0.92 (0.60,1.41) | 0.70 | 0.85 | 1.24 (0.91,1.68) | 0.17 | 0.58 |
| rs11003131 | TLR8 | 1.00 (0.63,1.60) | 0.99 | 0.99 | 1.47 (0.36,5.97) | 0.59 | 0.77 | 0.96 (0.59,1.55) | 0.87 | 0.87 | 1.03 (0.68,1.57) | 0.88 | 0.92 |
| rs5741880 | TLR7 | 0.75 (0.30,1.88) | 0.54 | 0.94 | 1.77 (0.42,7.52) | 0.44 | 0.68 | 0.47 (0.14,1.63) | 0.24 | 0.76 | 0.95 (0.50,1.82) | 0.88 | 0.92 |

**Supplementary Table 8.** Power analysis of the relationship between host SNPs in TLR7 (rs5741880), TLR8 (rs3764880) and vaccine response. Table shows the minimum of effect size that could achieve 80% power under current sample size

|  | TLR7 (rs5741880) | | | TLR8 (rs3764880) | | |
| --- | --- | --- | --- | --- | --- | --- |
|  | Current sample size | Absolute value of regression coefficient that could achieve 80% power | Current regression coefficient | Current sample size | Absolute value of regression coefficient that could achieve 80% power | Current regression coefficient |
| HAI titer ≥1:40 after vaccination for the three vaccine strains (H1N1, H3N2 and B) | | | | | | |
| Dominant model | 495 | 1.03 | -0.94 | 494 | 0.63 | -0.56 |
| Recessive model | 495 | 3.02 | 1.22 | 494 | 0.94 | -0.12 |
| Over-dominant model | 495 | 1.25 | -1.51 | 494 | 0.75 | -0.76 |
| Multiplicative model | 495 | 0.23 | -0.42 | 494 | 0.25 | -0.26 |
| Average increase of logarithm of geometric mean titer (GMT) after vaccination for the three vaccine strains (H1N1, H3N2 and B) | | | | | | |
| Dominant model | 492 | 1.38 | -1.17 | 491 | 0.72 | -0.42 |
| Recessive model | 492 | 2.77 | -0.76 | 491 | 1.15 | -0.02 |
| Over-dominant model | 492 | 1.52 | -1.27 | 491 | 0.95 | -0.62 |
| Multiplicative model | 492 | 0.86 | -0.78 | 491 | 0.27 | -0.19 |
| ≥4-fold rise in antibody after vaccination for the three vaccine strains (H1N1, H3N2 and B) | | | | | | |
| Dominant model | 492 | 1.30 | -0.69 | 491 | 0.68 | -0.31 |
| Recessive model | 492 | 2.09 | 0.30 | 491 | 0.91 | 0.24 |
| Over-dominant model | 492 | 1.74 | -1.17 | 491 | 0.94 | -0.76 |
| Multiplicative model | 492 | 0.76 | -0.33 | 491 | 0.21 | -0.09 |

**Supplementary Table 9.** Antibody response after influenza vaccination by vaccine strain

|  | Seasonal A(H1N1) | Seasonal A(H3N2) | B | p-value |
| --- | --- | --- | --- | --- |
| Antibody titer >= 40 |  |  |  |  |
| Pre-vaccination | 297/545 (54.5%) | 306/545 (56.1%) | 123/544 (22.6%) | < 0.001 |
| Post-vaccination | 503/536 (93.8%) | 515/536 (96.1%) | 386/535 (72.1%) | 0.002 |
| Average Increase of logarithm of geometric mean titer (GMT) after vaccination | 3.63 | 3.99 | 2.74 | < 0.001 |
| ≥4-fold rise in antibody after vaccination | 339/533 (63.6%) | 397/533 (74.5%) | 305/532 (57.3%) | 0.03 |
