# Supplementary material for: Impact of host genetic polymorphisms on response to inactivated influenza vaccine in children
Source: NPJ Vaccines. 2023 Feb 20;8:21. doi: 10.1038/s41541-023-00621-1 (PMC9940051; doi:10.1038/s41541-023-00621-1)
Supplement: Supplementary file 2 — Reporting summary [file 41541_2023_621_MOESM2_ESM.pdf]

nature portfolio

Corresponding author(s): Tim K. Tsang

Last updated by author(s): Jan 12, 2023

Reporting Summary

Nature Portfolio wishes to improve the reproducibility of the work that we publish. This form provides structure for consistency and transparency in reporting. For further information on Nature Portfolio policies, see our Editorial Policies and the Editorial Policy Checklist.

Statistics

For all statistical analyses, confirm that the following items are present in the figure legend, table legend, main text, or Methods section.

n/a	Confirmed	
☐	☒	The exact sample size (*n*) for each experimental group/condition, given as a discrete number and unit of measurement
☒	☐	A statement on whether measurements were taken from distinct samples or whether the same sample was measured repeatedly
☐	☒	The statistical test(s) used AND whether they are one- or two-sided<br>*Only common tests should be described solely by name; describe more complex techniques in the Methods section.*
☐	☒	A description of all covariates tested
☐	☒	A description of any assumptions or corrections, such as tests of normality and adjustment for multiple comparisons
☐	☒	A full description of the statistical parameters including central tendency (e.g. means) or other basic estimates (e.g. regression coefficient) AND variation (e.g. standard deviation) or associated estimates of uncertainty (e.g. confidence intervals)
☒	☐	For null hypothesis testing, the test statistic (e.g. *F*, *t*, *r*) with confidence intervals, effect sizes, degrees of freedom and *P* value noted<br>*Give P values as exact values whenever suitable.*
☒	☐	For Bayesian analysis, information on the choice of priors and Markov chain Monte Carlo settings
☒	☐	For hierarchical and complex designs, identification of the appropriate level for tests and full reporting of outcomes
☒	☐	Estimates of effect sizes (e.g. Cohen's *d*, Pearson's *r*), indicating how they were calculated

Our web collection on statistics for biologists contains articles on many of the points above.

Software and code

Policy information about availability of computer code

Data collection	no software is used
Data analysis	All statistical analyses were conducted using R version 3.6.1 (R Foundation for Statistical Computing, Vienna, Austria).

For manuscripts utilizing custom algorithms or software that are central to the research but not yet described in published literature, software must be made available to editors and reviewers. We strongly encourage code deposition in a community repository (e.g. GitHub). See the Nature Portfolio guidelines for submitting code & software for further information.

Data

Policy information about availability of data

All manuscripts must include a data availability statement. This statement should provide the following information, where applicable:

- Accession codes, unique identifiers, or web links for publicly available datasets
- A description of any restrictions on data availability
- For clinical datasets or third party data, please ensure that the statement adheres to our policy

The data that support the findings of this study are available on request from the corresponding author TKT. The data are not publicly available due to containing information that could compromise research participant privacy.

Human research participants

Policy information about studies involving human research participants and Sex and Gender in Research.

Reporting on sex and gender	there is no sex or gender based analysis
Population characteristics	General population in Hong Kong
Recruitment	Households with at least one child aged 6-17 who did not have any contraindications against injection of inactivated influenza vaccine including allergy or hypersensitivity to eggs or other substances contained in the vaccine. Age of household members were ranged from 0-90, and 47% of household members were male.
Ethics oversight	the Institutional Review Board of the University of Hong Kong and the Hong Kong Department of Health Ethics Committee.

Note that full information on the approval of the study protocol must also be provided in the manuscript.

Field-specific reporting

Please select the one below that is the best fit for your research. If you are not sure, read the appropriate sections before making your selection.

☒ Life sciences ☐ Behavioural & social sciences ☐ Ecological, evolutionary & environmental sciences

For a reference copy of the document with all sections, see nature.com/documents/nr-reporting-summary-flat.pdf

Life sciences study design

All studies must disclose on these points even when the disclosure is negative.

Sample size	his paper is a secondary analysis for data collected from two trials and follow-up study. The two community-based randomized controlled trials (RCTs) are aim to evaluate direct and indirect benefits of influenza vaccination. In the trial, for estimating direct vaccine efficacy, assuming conservatively that 10% of participants in the control arm would be infected with a prevalent influenza strain, inclusion of 800 subjects would have 75% power to detect a vaccine efficacy of ≥50%, with a 5% type I error rate. An unbalanced randomization scheme was proposed, where more participants were included in the intervention arm to enhance acceptability. For estimating indirect vaccine efficacy, recruiting the 2,000 (assuming an average household size of 3.5 = 1 participant + 2.5 household contacts) household members of the 800 study participants (1,200 in intervention arm, 800 in control arm) was anticipated to be sufficient to ensure high power to identify indirect vaccine effectiveness values of interest. While the infection risk of influenza in adults is typically lower than in children, we anticipated an attack rate of 5% in the control arm. The within-household correlation in infection risks was reported as 29% in a previous study. Allowing for within-household correlation, our study would have 77% power to detect indirect vaccine effectiveness of 40% or greater, with a 5% type I error rate.
Data exclusions	Participants provided no serum sample were excluded in the study, because they contributed no information for analyses.
Replication	Simulation study was conducted to ensure our inference is valid. Computer code is available for reproducing the results with the same data.
Randomization	This paper is a secondary analysis for data collected from two trials and follow-up study. In the trials, Randomization lists were prepared by a biostatistician (V. J. F). Eligible study participants were randomly allocated to the TIV or placebo group at a ratio of 3:2 using a random number generator (R software). A block-randomization sequence was generated, with randomly permuted block sizes of 5, 10, and 15
Blinding	This paper is a secondary analysis for data collected from two trials and follow-up study. Blinding of households and study nurses was achieved by identical repackaging of TIV/placebo into numbered syringes by a trained nurse not involved in vaccine administration. A research assistant who had no access to the randomization list allocated unique numbers to participating households based on their order of attendance and these were subsequently matched to vaccine packages. Allocation of TIV/placebo was concealed to participating households, study nurses, and laboratory staff, and was revealed to investigators only after completion of follow-up.

Reporting for specific materials, systems and methods

We require information from authors about some types of materials, experimental systems and methods used in many studies. Here, indicate whether each material, system or method listed is relevant to your study. If you are not sure if a list item applies to your research, read the appropriate section before selecting a response.

Materials & experimental systems

n/a	Involved in the study
☒	☐ Antibodies
☒	☐ Eukaryotic cell lines
☒	☐ Palaeontology and archaeology
☒	☐ Animals and other organisms
☒	☐ Clinical data
☒	☐ Dual use research of concern

Methods

n/a	Involved in the study
☒	☐ ChIP-seq
☒	☐ Flow cytometry
☒	☐ MRI-based neuroimaging
